# Effects of the Full Coverage Policy of Essential Medicines on Inequality in Medication Adherence: A Longitudinal Study in Taizhou, China

**DOI:** 10.3389/fphar.2022.802219

**Published:** 2022-02-03

**Authors:** Zhigang Guo, Liguang Zheng, Mengyuan Fu, Huangqianyu Li, Lin Bai, Xiaodong Guan, Luwen Shi

**Affiliations:** ^1^ Department of Pharmacy, Peking University School and Hospital of Stomatology, Beijing, China; ^2^ International Research Center for Medicinal Administration, Peking University, Beijing, China; ^3^ School of Pharmaceutical Sciences, Peking University, Beijing, China

**Keywords:** full coverage policy, free, essential medicines, inequality in medication adherence, concentration index, China

## Abstract

The full coverage policy for essential medicines (FCPEMs) was proposed and implemented in Taizhou city of Zhejiang Province, China, to promote equal access and adherence to medicines. This study aimed to examine the effects of FCPEMs on the income-related inequality in medication adherence among local patients with hypertension or diabetes. We collected electronic health records of patients with hypertension or diabetes of three districts of Taizhou from 2011 to 2016. As the implementation schedule of the FCPEMs varied across districts, we applied a retrospective longitudinal study design and assigned records from 1 year before to 3 years following the implementation of FCPEMs as baseline and follow-up data. We thus generated a dataset with 4-year longitudinal data. The concentration index (CI) and its decomposition method were employed to measure factors contributing to inequality in medication adherence and the role played by FCPEMs. The sample size rose from 264,836 at the baseline to 315,677, 340,512, and 355,676 by each follow-up year, and the proportion of patients taking free medicines rose from 17.6 to 25.0 and 29.8% after FCPEMs implementation. The proportion of patients with high adherence increased from 39.9% at baseline to 51.6, 57.2, and 60.5%, while CI decreased from 0.073 to −0.011, −0.029, and −0.035. The contribution of FCPEMs ranked at 2^nd^/13, 7^th^/13, and 2^nd^/13 after the implementation of FCPEMs. Changes in CI of medication adherence for every 2 years were −0.084, −0.018, and −0.006, and the contribution of FCPEMs was −0.006, 0.006, and 0.007, ranking at 2^nd^/13, 2^nd^/13, and 1^st^/13, respectively. Most changes in CI of medication adherence can be attributed to FCPEMs. The medication adherence of patients with hypertension or diabetes improved after the implementation FCPEMs in Taizhou, although inequality did not improve consistently. In general, FCPEMs could be a protective factor against income-related inequalities in access and adherence to medicines. Future research is needed to investigate the change mechanism and the optimal design of similar interventions.

## Introduction

Unequal access to medicines is a universal problem for health-care system reforms (Kolasa and Kowalczyk, 2016). It exacerbates a phenomenon well documented in the literature that people from lower income and minority groups experience higher health risks and are more vulnerable to medication costs (Fu et al., 2020; Pinto et al., 2020). The full coverage policy for medicines (FCPMs) is a policy strategy to promote equal access and adherence to medicines. Depending on specific contexts, FCPMs may also be known as “free,” “full reimbursement,” or “fee exemption” medicine policy. According to the Pharmaceutical Country Profiles by the World Health Organization, all 105 listed countries had implemented FCPMs to some extent. Among these countries, 54 had full coverage programs for any essential medicines (Wang et al., 2019). FCPMs is also increasingly studied and implemented in hypertension and diabetes interventions in the United States (Zheng et al., 2017; Ross-Degnan et al., 2020), Canada (Laba et al., 2020), Brazil (Emmerick et al., 2020), Spain (Puig-Junoy et al., 2016; Gonzalez et al., 2017), Madagascar (Garchitorena et al., 2017), India (Bose and Dutta, 2018), and Burkina Faso (Yaogo, 2017).

China has adopted FCPMs for antihypertensive and hypoglycemic medicines. By the end of 2020, 16 provinces had selected at least one district/county as the pilot area(s) to explore various policy strategies to manage hypertension and diabetes (Yu et al., 2013; Chen and Pan, 2019; Wang et al., 2019; Gong et al., 2020). In 2011, Taizhou, Zhejiang, was among the first cities to implement the full coverage policy for essential medicines (FCPEMs). The policy was set out to be as follows: 1) all nine districts/counties administered by Taizhou city were required to develop a full-coverage medication catalog between 2012 and 2013, specifying what medicines for hypertension and diabetes listed in the National Essential Medicines List of China (version 2012) would be covered in full. 2) Eligible patients could access medicines listed in this catalog without any costs, including drug, prescription, and related medical costs, at any primary care or designated facilities. 3) Physicians at primary care and designated facilities were responsible for evaluating selected medicines’ clinical benefits and appropriateness for patients, and adjusting the medication plan if clinical outcomes were poor. 4) As part of the basic public health services, China had been establishing health records and providing free chronic disease management services for every patient with hypertension and diabetes from the new healthcare reform in 2009 (Li et al., 2017; Lai et al., 2021). All hypertension and diabetes patients using medicines covered must be enrolled in chronic disease management services and have their medication and disease management plan incorporated into health records.

Studies indicated that FCPMs and FCPEMs could reduce medicine-related costs for patients, improve affordability and accessibility of covered medicines, and promote medication adherence (Choudhry et al., 2011; Kolasa and Kowalczyk, 2016; Wang et al., 2019). It can also help with disease prevention and control (Yu et al., 2013) and help in achieving more economical benefits for the society (Ito et al., 2015). There is emerging evidence that implementation of FCPMs and FCPEMs would favorably benefit vulnerable populations in accessing medications (Yu et al., 2013; Kolasa and Kowalczyk, 2016; Gonzalez et al., 2017; Yaogo, 2017), though this is better supported by further investigations. Therefore, we aimed to analyze the change in medication adherence before and after the implementation of FCPEMs in Taizhou and to explore the policy’s effects on income-related inequality in its first years’ implementation.

## Methods

### Settings

Taizhou is a prefecture-level city with a total area of 10,050 km^2^ in Zhejiang Province, located in the central area of the Yangtze River Delta in China. The city administers three urban districts (Jiaojiang, Huangyan, and Luqiao), three county-level cities (Linhai, Wenling, and Yuhuan), and three counties (Tianhai, Xianju, and Sanmen). In 2016, Taizhou had a population of 5.2 million, with 9.1% of them older than 65 years and 19.3% aged 45–64 years. The annual disposable income per capita was 5321.5 dollars. The average number of health professionals, hospital beds, physicians, and nurses for every thousand residents was 6.8, 4.5, 2.8, and 2.7, respectively (Taizhou Bureau of Statistics, 2017).

### Data Source and Design

In 2017, we collected electronic health records of patients with hypertension or diabetes from 2011 to 2016, which included patients’ demographics and regular follow-up data (e.g., medicines prescribed, level of medication adherence, and health behaviors such as smoking and drinking) (Li et al., 2017). We used a retrospective longitudinal study design to analyze the impact of FCPEMs. As the policy was implemented in the nine districts/counties with different schedules, patient records collected 12 months prior to policy implementation were regarded as baseline data. Records of 1–12 months, 13–24 months, and 25–36 months after policy implementation were regarded jointly as follow-up data. Thus, we generated a set of 4-year retrospective longitudinal data. Due to system upgrades, only data of Huangyan district, Linhai city, and Wenling city were unabridged and eligible over the study period. Therefore, the analysis of this study was limited to patients residing in these three areas. The policy implementation schedule and the medicine catalog for each area are shown in Supplementary Appendix Table S1.

### Measures and Determinants

According to the Chronic Disease Management Services of China (Lai et al., 2021), family physicians should administer follow-up surveys regularly (every 3 months) to monitor disease status. In Taizhou, medication adherence would be assessed in follow-up surveys and be translated into three categories: “*regular medication use*,” “*irregular medication use*,” and “*taking no medication*.” Patients taking medicines strictly following the prescribed schedule were assigned “*regular medication use*,” and patients reporting no medicine use was were assigned “*taking no medication*.” The remaining would be categorized into “*irregular medication use*.” In our analysis, “*regular medication use*” was treated as high adherence (computed as 1), and “*irregular medication use*” and “*taking no medication*” were regarded as poor adherence (computed as 0).

If the patient had used the medicines covered under FCEMPs in the given year, they would be categorized as *FCPEM covered* (computed as 1), otherwise as *not covered* (computed as 0).

Based on a previous research (Marsicano et al., 2015; Cathbert, 2019; Sipos et al., 2021), our analysis also included the following regular follow-up variables which might impact medication adherence (Table 1): gender, age, income group (the low-/middle-/high-income group with one-third of population by rank of the annual household income), *hukou* status, residential area, marital status, employment status, education level, medical insurance, smoking, drinking, and disease type.

**TABLE 1:** Characteristics of the study population in the 4 years.

Variable	Description	Baseline (*n* = 264,836)	First year (*n* = 315,677)	Second year (*n* = 340,512)	Third year (*n* = 355,676)
Dependent variable	High adherence, %	39.9	51.6	57.2	60.5
	Poor adherence, %	60.1	48.4	42.8	39.5
Policy	FCPEMs covered, %	—	17.6	25.0	29.8
	Not covered, %	—	82.4	75.0	70.2
Gender	Female, %	61.1	60.0	59.6	60.3
	Male, %	38.9	40.0	40.4	40.7
Age (years)	0–64, %	50.0	50.1	48.8	47.1
	65+, %	50.0	49.9	51.2	52.9
Income group	Low, %	33.3	33.3	33.3	33.3
	Middle, %	33.3	33.3	33.3	33.3
	High, %	33.3	33.3	33.3	33.3
Hukou	Non-agricultural, %	2.3	2.6	2.9	2.9
	Agricultural, %	97.7	97.4	97.1	97.1
Residential terrain	Plain area, %	72.9	73.1	73.7	74.2
	Mountainous area, %	27.1	26.9	26.2	25.8
Marital status	Unmarried, %^a^	18.0	16.9	16.2	15.6
	Married, %^b^	82.0	83.1	83.8	84.4
Employment status	Employed, %	13.2	13.7	14.0	14.2
	Unemployed, %	86.8	86.3	86.0	85.8
Educational level	Illiterate and semiliterate, %	40.6	38.4	36.9	35.6
	Primary school, %	42.3	42.4	42.9	43.3
	Junior middle school, %	14.5	16.0	16.8	17.7
	High school and above, %	2.6	3.2	3.4	3.5
Medical insurance	None, %	4.9	5.1	5.1	5.3
	URRBMI, %^c^	92.0	91.4	91.1	90.5
	UEBMI and CMI, %^d^	3.1	3.5	3.8	4.1
Smoke	No, %	85.3	84.5	84.9	84.4
	Yes, %	14.7	15.5	15.1	15.6
Drink	No, %	92.8	92.3	92.3	91.2
	Yes, %	7.2	7.7	7.7	8.8
Disease	Hypertension, %	81.5	80.4	79.7	78.7
	Diabetes, %	18.5	19.6	20.3	21.3

a Note: Including married and remarried.

b Including unmarried, divorced, and widowed.

c Urban Rural Resident Basic Medical Insurance.

d Urban Employee Basic Medical Insurance and Commercial Medical Insurance.

### Statistical Analysis

The concentration index (CI) and its decomposition recommended by Wagstaff and Van Doorslaer were applied to analyze the inequality of medication adherence and the contribution of the determinant factors (Fu et al., 2020; Lai et al., 2021). The calculation formula of CI is as follows:

$$CI=\frac{2}{\mu }cov\left(y_{i},r_{i}\right),$$ (1)

where $y_{i}$ denotes medication adherence (0 or 1), μ is the mean of $y_{i}$ , and $r_{i\;}$ is the fractional rank of the individual in the economic distribution. The value of CI ranges from −1 to 1, while the smaller absolute value represents higher equality. Thus, 0 implies perfect equality. A positive value signifies a pro-rich effect and a negative value signifies pro-poor effects. The model of the decomposition of CI (Zhao et al., 2021) is as follows:

$$CI=\underset{k}{\sum }\left(\frac{\;\gamma_{k}{\bar{X}}_{k}}{\mu }\right)CI_{K}+\;\frac{GCI_{\varepsilon }}{\mu },$$ (2)

where $\gamma_{k}$ , ${\bar{X}}_{k}$ , and $CI_{K}$ are the marginal effect, mean, and *CI* of the independent variables, respectively. $\;\frac{GCI_{\varepsilon }}{\mu }$ represents the residual error team. $\left(\frac{\;\gamma_{k}{\bar{X}}_{k}}{\mu }\right)$ denotes the elasticity between $CI_{y}$ and $CI_{K}$ , and $\left(\frac{\;\gamma_{k}{\bar{X}}_{k}}{\mu }\right)CI_{K}$ is the contribution of the determinant factors. So $\left(\frac{\;\gamma_{k}{\bar{X}}_{k}}{\mu }\right)CI_{K}/CI_{y}$ is the rate of contribution.

In order to understand the effect of FCPEMs on medication adherence, the Oaxaca-type decomposition (Pulok et al., 2020) is applied, and the equation is as follows:

$$\begin{array}{l} CI_{y\left(t\right)}-CI_{y\left(t-1\right)}=\underset{k}{\sum }{\text{η}}_{x\left(t\right)}\left(CI_{x\left(t\right)}-CI_{x\left(t-1\right)}\right)+\underset{k}{\sum }CI_{x\left(t-1\right)}\left({\text{η}}_{x\left(t\right)}-{\text{η}}_{x\left(t-1\right)}\right),\\ \Delta \left(\frac{GCI_{\varepsilon }}{\mu_{t}}\right)=\underset{k}{\sum }{\text{η}}_{x\left(t\right)}\Delta CI_{x}+\underset{k}{\sum }CI_{x\left(t-1\right)\;}\Delta {\text{η}}_{x}+\Delta \left(\frac{GCI_{\varepsilon }}{\mu_{t}}\right), \end{array}$$ (3)

where η is the elasticity and calculated as $\left(\frac{\;\gamma_{k}{\bar{X}}_{k}}{\mu }\right)$ . The ${\text{η}}_{x\left(t\right)}\Delta CI_{x}$ represents the changes of the socioeconomic inequality in determinant factors, and $CI_{x\left(t-1\right)\;}\Delta {\text{η}}_{x}$ measures the changes of sensitivity between $CI_{y}$ and $CI_{K}$ . All statistical analyses were performed using STATA software version 14.0.

## Results

### Characteristics of Study Population

Table 1 shows the characteristics of the study population from baseline to 3 years after the implementation of FCPEMs. The sample size rose from 264,836 at baseline to 315,677, 340,512, and 355,676 patients by each follow-up year, of which 81.5, 80.4, 79.7, and 78.7% were patients with hypertension, respectively. Over the study period, the proportion of patients with high adherence increased from 39.9% to 51.6, 57.2, and 60.5%. The proportion of patients taking free medicines also increased with time, from 17.6 to 25.0% and 29.8% after the FCPEMs.

### Description in Medication Adherence and Its Concentration Index

Table 2 demonstrates changes in medication adherence, stratified by income, and CI of medication adherence over the 4 years. Patients from all income groups experienced varying degrees of improvement in medication adherence. Patients from the low-income group experienced largest improvement (from 33.2% to 53.0, 61.2, and 65.4%), followed by patients from the middle-income group (from 42.6% to 51.6, 56.6, and 59.3%). Patients from the high-income group experienced the smallest increase in the adherence rate, from 44.6% to 50.2, 53.6, and 56.5%. CI of medication adherence decreased from 0.073 to −0.011, −0.029, and −0.035, showing an increasing favorability of FCPEMs’ effects toward the lower income.

**TABLE 2:** Medication adherence and CI of the 4 years.

Medication adherence	Baseline (*n* = 264,836)	First year (*n* = 315,677)	Second year (*n* = 340,512)	Third year (*n* = 355,676)
Proportion of high adherence				
Low-income group	33.2%	53.0%	61.2%	65.4%
Middle-income group	42.6%	51.6%	56.6%	59.3%
High-income group	44.6%	50.2%	53.6%	56.5%
CI	0.073	−0.011	−0.029	−0.035

### Decomposition of Inequality in Medication Adherence

Table 3 presents the elasticity and the rate of contribution of determinants on CI of medication adherence over the 4 years. During the follow-up period, the elasticity of the policy increased from 0.103 to 0.131, 0.141, respectively, implying that the positive association between CI of the policy and medication adherence strengthened overtime. The rate of the contribution of the policy to medication adherence changed from 54.792 to 1.223% and −19.092% by the end of each follow-up year, respectively. Its ranking dropped from 2^nd^/13 at the end of the first follow-up year to 7^th^/13 by the second year and climbed back to 2^nd^/13 by the end of the follow-up period. Meanwhile, the CI of medication adherence were −0.011, −0.029, and −0.035, respectively. FCPEMs was a pro-poor contributing factor at the initial 2 years of implementation and a pro-rich contribution factor in the third year.

**TABLE 3:** Decomposition of CI in medication adherence over the 4 years.

Determinant	Baseline (*n* = 264,836)		First year (*n* = 315,677)		Second year (*n* = 340,512)		Third year (*n* = 355,676)	
	Elast^1^	R of cont^2^ (%)	Elast^1^	R of cont^2^ (%)	Elast^1^	R of cont^2^ (%)	Elast^1^	R of cont^2^ (%)
Policy (Ref: control)								
Free medicines	0.000	0.000%	0.103	54.792%	0.131	1.223%	0.141	−19.092%
Gender (Ref: female)								
Male	−0.012	−0.533%	−0.008	2.155%	−0.008	0.731%	−0.004	0.254%
Age (Ref: 0–64 years)								
65+	0.046	−4.748%	0.045	28.947%	0.038	9.038%	0.036	6.206%
Income group (Ref: low)								
Middle	0.068	3.319%	−0.026	6.806%	−0.050	4.711%	−0.055	3.161%
High	0.086	75.918%	−0.030	175.906%	−0.069	156.103%	−0.074	139.946%
Hukou (Ref: non-agricultural)								
Agriculture	−0.156	1.735%	−0.130	−10.632%	−0.201	−7.496%	−0.140	−4.359%
Residential terrain (Ref: plain area)								
Mountainous area	−0.010	−0.386%	−0.020	2.863%	−0.020	0.588%	−0.023	0.564%
Marital status (Ref: unmarried)								
Married	−0.005	−0.144%	0.010	−1.842%	0.011	−0.738%	0.000	0.002%
Employment status (Ref: employed)								
Unemployed	−0.021	1.119%	−0.128	−44.608%	−0.124	−16.909%	−0.086	−9.640%
Educational level (Ref: illiterate and semiliterate)								
Primary school	−0.008	0.056%	0.018	−0.140%	0.022	−0.024%	0.023	−0.137%
Junior middle school	−0.002	−0.024%	0.011	0.711%	0.013	0.386%	0.013	0.691%
High school and above	0.001	−0.096%	0.003	2.301%	0.003	0.796%	0.003	0.635%
Medical insurance (Ref: none)								
URRBMI	−0.084	0.596%	−0.098	−2.160%	−0.040	−0.479%	−0.031	−0.363%
UEBMI and CMI	0.002	−0.013%	0.000	0.023%	0.005	0.089%	0.006	0.389%
Smoke (Ref: no)								
Yes	−0.013	0.949%	−0.012	−4.142%	−0.012	−0.725%	−0.012	−0.622%
Drink (Ref: no)								
Yes	0.002	0.065%	−0.001	−0.128%	−0.005	−0.156%	−0.004	−0.249%
Disease (Ref: hypertension)								
Diabetes	−0.082	−1.060%	−0.100	−4.332%	−0.109	−0.275%	−0.108	0.001%

Note: Elast^1^: elasticity; R of Cont^2^: rate of contribution.

### Change in the Decomposition of CI in Medication Adherence

Table 4 presents the contribution of all determinants in changes medication adherence every 2 years, with an Oaxaca-type decomposition. The change in CI in medication adherence was −0.084, −0.018, and −0.006 for every 2 years from baseline to 3 years after FCPEMs, of which the contribution of the FCPEMs was −0.0061, 0.0057, and 0.0070 and it ranked at the 2^nd^/13, 2^nd^/13, and 1^st^/13 place, respectively. From the results of the Oaxaca-type decomposition, the contribution of the policy was mainly attributable to the changes in CI of FCPEMs (−0.0061, 0.0074, and 0.0070) and experienced a minor impact by the changes of sensitivity (0.0000, −0.0017, and 0.0000).

**TABLE 4:** Oaxaca decomposition of CI in medication adherence over the 4 years.

Determinant	Baseline–first year			First–second year			Second–third year		
	ηΔCI ^1^	${\text{CI}}_{\;}\Delta \text{η}$ ^2^	ΔCont ^3^	ηΔCI ^1^	${\text{CI}}_{\;}\Delta \text{η}$ ^2^	ΔCont ^3^	ηΔCI ^1^	${\text{CI}}_{\;}\Delta \text{η}$ ^2^	ΔCont ^3^
Policy (Ref: control)									
Free medicines	−0.0061	0.0000	−0.0061	0.0074	−0.0017	0.0057	0.0070	0.0000	0.0070
Gender (Ref: female)									
Male	0.0000	0.0001	0.0002	0.0000	0.0000	0.0000	0.0000	0.0001	0.0001
Age (Ref: 0–64 years)									
65+	0.0002	0.0001	0.0003	0.0001	0.0005	0.0006	0.0003	0.0002	0.0004
Income group (Ref: low)									
Middle	0.0002	−0.0034	−0.0032	0.0001	−0.0007	−0.0006	0.0004	−0.0001	0.0003
High	−0.0001	−0.0749	−0.0750	−0.0001	−0.0254	−0.0255	−0.0003	−0.0034	−0.0037
Hukou (Ref: non-agricultural)									
Agriculture	0.0001	−0.0002	−0.0001	0.0003	0.0006	0.0010	0.0000	−0.0007	−0.0006
Residential terrain (Ref: plain area)									
Mountainous area	0.0002	−0.0003	0.0000	0.0002	0.0000	0.0001	0.0000	0.0000	0.0000
Marital status (Ref: unmarried)									
Married	0.0000	0.0003	0.0003	0.0000	0.0000	0.0000	0.0000	−0.0002	−0.0002
Employment status (Ref: employed)									
Unemployed	−0.0001	0.0042	0.0041	0.0001	−0.0002	−0.0001	0.0000	−0.0015	−0.0015
Educational level (Ref: illiterate and semiliterate)									
Primary school	0.0001	−0.0001	0.0000	0.0000	0.0000	0.0000	0.0000	0.0000	0.0000
Junior middle school	−0.0002	0.0001	−0.0001	0.0000	0.0000	0.0000	−0.0001	0.0000	−0.0001
High school and above	−0.0001	−0.0001	−0.0002	0.0001	0.0000	0.0000	0.0000	0.0000	0.0000
Medical insurance (Ref: none)									
URRBMI	−0.0003	0.0001	−0.0002	0.0000	−0.0001	−0.0001	0.0000	0.0000	0.0000
UEBMI and CMI	0.0000	0.0000	0.0000	0.0001	−0.0002	0.0000	−0.0001	0.0000	−0.0001
Smoke (Ref: no)									
Yes	−0.0002	−0.0001	−0.0002	−0.0003	0.0000	−0.0002	0.0000	0.0000	0.0000
Drink (Ref: no)									
Yes	0.0000	−0.0001	0.0000	0.0000	0.0001	0.0000	0.0000	0.0000	0.0000
Disease (Ref: hypertension)									
Diabetes	0.0014	−0.0002	0.0013	−0.0004	0.0000	−0.0004	−0.0001	0.0000	−0.0001

Note: ηΔCI : ${\text{η}}_{\text{x}\left(\text{t}\right)}\Delta {\text{CI}}_{\text{x}}={\text{η}}_{\text{x}\left(\text{t}\right)}\left({\text{CI}}_{\text{x}\left(\text{t}\right)}-{\text{CI}}_{\text{x}\left(\text{t}-1\right)}\right);$ ${\text{CI}}_{\;}\Delta {\text{η:CI}}_{\text{x}\left(\text{t}-1\right)\;}\Delta {\text{η}}_{\text{x}}={\text{CI}}_{\text{x}\left(\text{t}-1\right)}\left({\text{η}}_{\text{x}\left(\text{t}\right)}-{\text{η}}_{\text{x}\left(\text{t}-1\right)}\right);$ ΔCont : $\text{η}\Delta {\text{CI}+\text{CI}}_{\;}\Delta \text{η}={\text{η}}_{\text{x}\left(\text{t}\right)}{\text{CI}}_{\text{x}\left(\text{t}\right)}-{\text{η}}_{\text{x}\left(\text{t}-1\right)}{\text{CI}}_{\text{x}\left(\text{t}-1\right)}$

## Discussion

This study examined income-related inequalities in medicine adherence using a retrospective longitudinal study design and a sample of patients with hypertension or diabetes in Taizhou, China. We identified that FCPEMs promoted overall medication adherence and favorably benefited patients from the low-income group over patients from other income groups.

Our study results are consistent with similar studies in a sense that medication adherence among patients improved after the implementation of FCPEMs and that largest improvement was observed in low-income population. With an increasing proportion of patients taking covered medicines by each year, medication adherence of the general population improved. In other studies of FCPMs, low-income patients who converted from poor to high medication adherence contributed to an increase of 7.8% in medication adherence to statins (Gonzalez et al., 2017) and increased twice more than the high-income patients with diabetes (Ross-Degnan et al., 2020).

Although the income-related inequality in medication adherence did not show consistent improvement over the follow-up period, FCPEMs was effective in improving the inequality in medication adherence. Over the study period, the inequality of medication adherence improved in the first 2 years, but the outcome was conversed in the latter 2 years. In this process, FCPEMs helped in mitigating the changes of inequality. As two studies showed, FCPMs for cardiovascular disease and cancer reduced racial and ethnic disparities in medicines between white and non-white patients after an episode of myocardial infarction (Choudhry et al., 2014; Cole et al., 2020). For the exacerbated inequality, income group (high) was still the greatest barrier to realize equality, and FCPEMs became a positive element against it. As many studies suggest, medication non-adherence is significantly associated with low income (Sunny et al., 2020; Riley et al., 2021). Our study shows that FCPEMs could be a protecting factor against this income-related inequality in medication adherence experienced by low-income patients with hypertension and diabetes. This echoes a Targeted Poverty Alleviation program in China demonstrating that FCPMs could protect low-income patients against heavy burden of medicine costs (Chen and Pan, 2019).

However, due to the comprehensive impact of various factors, FCPEMs not always favorably benefited low-income patients. In our study, we knew change in CI of FCPEMs was the main contributor. The proportion of patients taking free medicines of different groups was thus the key to achieving sustainable equality in access and adherence to covered medicines. However, factors contributing to uptake of free medicines varied. One study showed that lower medication adherence among patients from high-income groups could result from a mismatch of free medicines with their health needs (Marsicano et al., 2015). Another study showed that age was a promoting factor for the uptake of free medicines; 40% of the older adults used free medicines to ameliorate their burden of medicine costs (Tavares et al., 2016). In this study, we found that the uptake of free medicines might be positively associated with an increase in free medicines covered by the catalog (Supplementary Appendix Table S1). We can reasonably conclude that the more health needs the medicines covered under FCPMs could meet, the more likely FCPMs could improve inequality in accessing and adherence to medicines. The geographical accessibility of designated health facilities (Emmerick et al., 2017), qualification of health professionals and pharmacists at these facilities (Zombre et al., 2017; Varas-Doval et al., 2020), and support from families and social networks (Tavares et al., 2016; Zhou et al., 2021) were also critical factors to consider.

It is also worth noting that long-term longitudinal data are necessary for evaluating the inequality in medication adherence and revising the FCPMs programs accordingly. If we only used 1-year data in this study after FCPEMs, the results and conclusions would be different or opposite. Meanwhile, medication adherence is influenced by many factors, thus it would need a comprehensive reform to improve inequality (Fosse-Edorh et al., 2015; Kolasa and Kowalczyk, 2016). During the reform, long-term longitudinal data were important for exhibiting the dynamic results (Zombre et al., 2017) and adjust the intervention precisely, such as the FCPEMs and FCPMs. All these highlighted the value of 4-year longitudinal data used by this study.

Nevertheless, our study has several limitations. First, the prevalence of hypertension and diabetes among residents registered in the local EMR database was 11.2 and 3.2%, respectively, in 2016, which were lower than the disease prevalence shown by epidemiological data (Li, 2021; Li et al., 2021; Xia et al., 2021). This implies a participant bias and that our sample might not be fully representative of the general population residing in Taizhou. Second, our measurement of the medication adherence was also subject to recall bias and administration bias as health records and follow-up surveys were collected by medical staffs, who might have varying degrees of training in administering surveys. Third, our results might have been affected by missing data and influence factors in the database, such as comorbidities. We made controls for this by aggregating all medical records of a patient within one calendar year into an annual data input. Patients with missing data after data aggregation were excluded from our study. Last, our baseline covered only 1 year as the database for chronic disease management was only established since 2011, and data prior to the year were inaccessible. This study schedule might influence the interpretation of our longitudinal data. However, we identified no other significant policy during local investigations, and our result that FCPEMs was a major driver for improvements in medication adherence still holds.

## Conclusion

In conclusion, overall medication adherence of the study population improved after the implementation of FCPEMs in Taizhou, and the low-income population experienced the largest increase in adherence to medicines. Interventions in the future should look into designing an optimal medicine catalog by first investigating the health needs of local residents to promote intervention uptake.

## Data Availability

The original contributions presented in the study are included in the article/Supplementary Material; further inquiries can be directed to the corresponding author.
