# Supplementary material for: Effects of the Full Coverage Policy of Essential Medicines on Inequality in Medication Adherence: A Longitudinal Study in Taizhou, China
Source: Front Pharmacol. 2022 Feb 3;13:802219. doi: 10.3389/fphar.2022.802219 (PMC8850774; doi:10.3389/fphar.2022.802219)
Supplement: Supplementary file 1 [file Table1.DOCX]

**Effects of the Full Coverage Policy of Essential Medicines on inequality in medication adherence: a longitudinal study in Taizhou, China**

**Appendix 1**

**Table1 The implementation time and medicines of the FCEMPs**

| District | Time | Antihypertensive medicines | Hypoglycemic medicines |
| --- | --- | --- | --- |
| Huangyan | 2012.06 | Captopril Tablets, Indapamide Tablets | Metformin Hydrochloride Tablets, Glipizide Tablets |
|  | 2013.04 | Supplementary: Telmisartan Tablets, Nitrendipine Tablets, Compound Reserpine Tablets, Zhenju antihypertensive tablets | Supplementary: Gliclazide Tablets |
| Linhai | 2013.10 | Captopril Tablets, Compound Reserpine Tablets, Indapamide Tablets, Nifedipine Tablets | Metformin Hydrochloride Tablets, Glipizide Tablets |
|  | 2015.08 | Supplementary: Amlodipine Besylate Tablets, Nifedipine Sustained-release Tablets, Metoprolol Tartrate Tablets, Zhenju antihypertensive tablets | Supplementary: Gliclazide Sustained-release Tablets |
| Wenling | 2013.02 | Captopril Tablets, Compound Reserpine Tablets, Hydrochlorothiazide Tablets, Amlodipine Besylate Tablets | Glipizide Tablets, Metformin Hydrochloride Enteric-coated Tablets |
|  | 2015.05 | Supplementary: Indapamide Tablets, Metoprolol Tartrate Tablets | None |
